# Histopathological Changes in the Liver, Heart and Kidneys Following Malayan Pit Viper (*Calloselasma rhodostoma*) Envenoming and the Neutralising Effects of Hemato Polyvalent Snake Antivenom

**DOI:** 10.3390/toxins14090601

**Published:** 2022-08-30

**Authors:** Wipapan Khimmaktong, Nazmi Nuanyaem, Nissara Lorthong, Wayne C. Hodgson, Janeyuth Chaisakul

**Affiliations:** 1 Division of Health and Applied Sciences, Faculty of Science, Prince of Songkla University, Songkhla 90110, Thailand; 2 Monash Venom Group, Department of Pharmacology, Biomedical Discovery Institute, Monash University, Melbourne, VIC 3800, Australia; 3 Department of Pharmacology, Phramongkutklao College of Medicine, Ratchathewi, Bangkok 10400, Thailand

**Keywords:** Malayan pit viper, snake, venom, cardiac, hepatic, renal, antivenom, histopathological

## Abstract

*Calloselasma rhodostoma* (Malayan pit viper) is a medically important snake species that is widely distributed across Southeast Asia. Systemic coagulopathy causing severe haemorrhage and local tissue injury is commonly observed following *C. rhodostoma* envenoming. However, nephrotoxicity and congestive heart failure were previously reported in a patient who had a long length of hospital stay. In this study, we determined the effect of *C. rhodostoma* envenoming on cardiovascular disturbances and the associated morphological changes in the liver, heart and kidneys using animal models. We also evaluated the efficacy of Hemato polyvalent antivenom (HPAV; Queen Saovabha Memorial Institute (QSMI) of the Thai Red Cross Society, Thailand) in neutralising the histopathological effects of *C. rhodostoma* venom. The intravenous (i.v.) administration of *C. rhodostoma* venom (1000 µg/kg) caused a rapid decrease in mean arterial pressure (MAP) followed by complete cardiac collapse in anaesthetized rats. Moreover, the intraperitoneal (i.p.) administration of *C. rhodostoma* venom (11.1 mg/kg; 3 × LD_50_) for 24 h caused cellular lesions in the liver and heart tissues. *C. rhodostoma* venom also induced nephrotoxicity, as indicated by the presence of tubular injury, interstitial vascular congestion and inflammatory infiltration in the whole area of the kidney. The administration of HPAV, at manufacturer-recommended doses, 15 min prior to or after the addition of *C. rhodostoma* venom reduced the extent of the morphological changes in the liver, heart and kidneys. This study found that experimental *C. rhodostoma* envenoming induced cardiovascular disturbances, hepatotoxicity and nephrotoxicity. We also highlighted the potential broad utility of HPAV to neutralise the histopathological effects of *C. rhodostoma* venom. The early delivery of antivenom appears capable of preventing envenoming outcomes.

## 1. Introduction

Snakebite envenoming is a major contributor to morbidity and mortality in the rural communities of sub-Saharan Africa, South Asia and Southeast Asia [1,2]. Annually, it is estimated that snake envenoming results in 81,000–138,000 deaths worldwide. In addition, snake envenoming is also responsible for permanent physical and psychological disabilities, including blindness, amputation and post-traumatic stress disorders [2]. In Thailand, the National Health Security Office (NHSO) reported 7.9 snakebite cases per 100,000 people in 2017. Five species of venomous snakes, i.e., monocled cobra (*Naja kaouthia*), Malayan krait (*Bungarus candidus*), Russell’s viper (*Daboia siamensis*), green pit viper (*Trimeresurus* spp.) and Malayan pit viper (*Calloselasma rhodostoma*), have been classified as category 1 venomous snakes, which cause high mortality and morbidity rates in the Public Health System of Thailand [3].

The Malayan pit viper (*C.* *rhodostoma*, subfamily Crotalinae, formerly known as *Angkistrodon rhodostoma*) is the species responsible for the most cases of envenoming in Thailand, accounting for 38% of bites [3]. *C. rhodostoma* has also been recognised as a species that causes high levels of morbidity, disability and mortality in Cambodia, the Indonesian Islands of Java and Madura, Peninsular Malaysia, Myanmar and Vietnam [2,3,4]. A number of studies have reported the basic proteomic profile of *C. rhodostoma* venom and indicated the presence of phospholipase A_2_ (PLA_2_), snake venom metalloproteinases (SVMPs), flavin monoamine oxidase and serine protease toxin families [5,6,7]. In addition, aminopeptidase, glutaminyl-peptide cyclotransferase and ankyrin repeats were recently identified in Malaysian *C. rhodostoma* venom [8].

The most significant clinical manifestations following *C. rhodostoma* envenoming are coagulopathy resulting in petechiae, epistaxis, haematuria and haemoptysis and consumption coagulopathy, which may result in death due to intracranial haemorrhage [3,9]. Local painful swelling and tissue necrosis at the bite-site are also commonly observed [4]. The local tissue damage is likely to be due to the effects of the myotoxic PLA_2_ in the venom. Snake venom PLA_2_ causes the disruption of plasma membrane integrity and sarcolemma damage, resulting in an influx of Ca^2+^ into the cytoplasm [10]. Moreover, the increase in intracellular Ca^2+^ also induces myofilament hypercontraction and mitochondrial dysfunction, leading to irreversible muscle damage and tissue necrosis [11,12].

In addition to hemodynamic disturbances, envenomings by medically important Asian vipers, e.g., Russell’s viper (*Daboia* spp.), green pit viper (*Trimersurus* spp.), hump-nosed pit viper (*Hypnale* spp.) and saw-scaled viper (*Echis* spp.), can induce nephrotoxicity, which is characterized by haematuria, tubular necrosis and acute renal failure [13,14]. Interestingly, a ten year retrospective study of *C. rhodostoma* envenoming in southern Thailand indicated that acute kidney injury (AKI) and congestive heart failure occurred in some patients envenomed by this species [15,16]. One death was reported due to complications associated with rhabdomyolysis after full recovery from systemic effects [16]. The administration of antivenom is the primary treatment strategy for victims of snake envenoming. In Thailand, The Queen Saovabha Memorial Institute (QSMI; Thai Red Cross Society, Bangkok, Thailand) produces two antivenoms that can be used for the treatment of *C. rhodostoma* envenoming. These are a monovalent antivenom, which only contains polyclonal antibodies derived from equine plasma hyperimmunized with *C. rhodostoma* venom, and a polyvalent antivenom, which comprises antibodies sourced from animals immunized with *C. rhodostoma* venom and venoms from other medically important haematotoxic snake species.

Although previous studies have proven the effectiveness of snake antivenom from QSMI in neutralizing circulating venom and reversing systemic symptoms [17,18], there are still clinical controversies regarding the risk of hypersensitivity and the high amounts of antivenom required to reverse nephrotoxicity following *C. rhodostoma* envenoming [9,15]. Moreover, the administration of monovalent antivenom at three times higher than the recommended therapeutic concentration was required to prevent nephrotoxicity following envenoming by eastern Russell’s vipers (*Daboia siamensis*) [19]. In the present study, we examined the effects of experimental *C. rhodostoma* envenoming in mice on the kidneys, liver and heart in the absence and presence of Hemato polyvalent antivenom (HPAV) using light microscopy and transmission electron microscopy (TEM). An evaluation of cardiovascular activity, i.e., the effects on mean arterial pressure (MAP) and heart rate, was also carried out in the anaesthetized rats.

## 2. Results

### 2.1. Effect of the Intravenous Administration of C. rhodostoma Venom on Anaesthetized Rats

**Figure 1:**
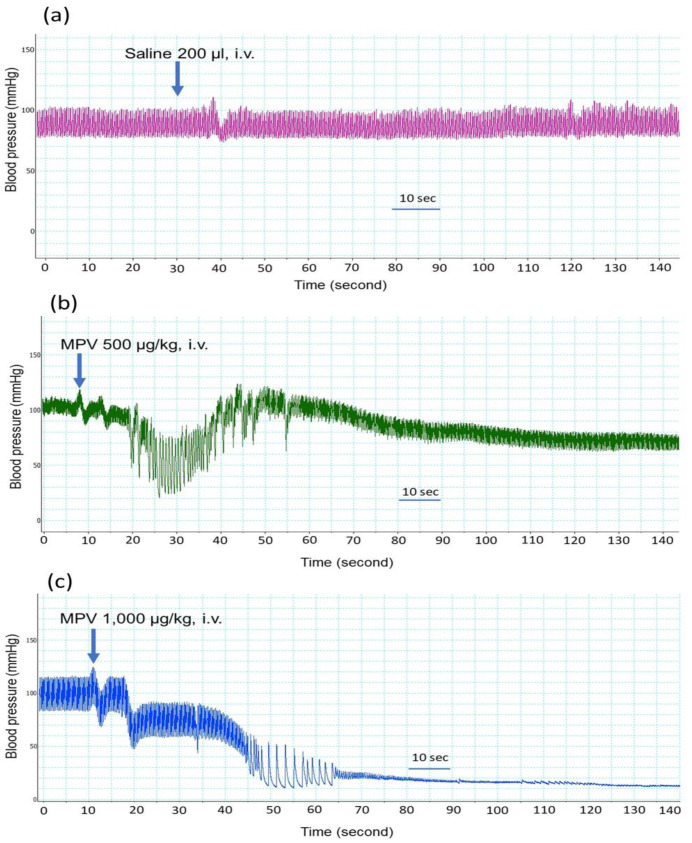
Traces showing the effect of the intravenous (i.v.) administration of (**a**) saline 200 µL, (**b**) *C. rhodostoma* venom (MPV) 500 µg/kg or (**c**) *C. rhodostoma* venom (MPV) 1000 µg/kg on the blood pressure (mmHg) of anaesthetized rats.

The intravenous administration of saline (200 µL) did not affect the blood pressure or heart rate of anaesthetized rats (Figure 1a). The administration of *C. rhodostoma* venom at doses between 200 and 400 µg/kg (*n* = 2; data not shown) did not cause significant changes in blood pressure and heart rate compared to the administration of saline.

**Figure 2:**
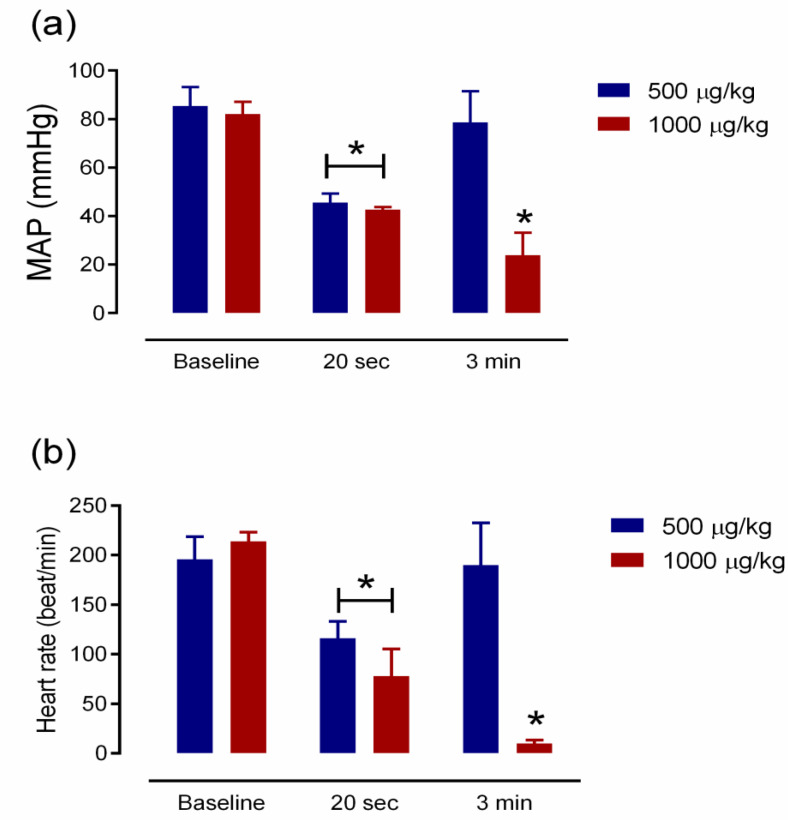
(**a**) Mean arterial pressure (MAP) before (baseline) and following the administration of *C. rhodostoma* venom (500–1000 µg/kg; i.v.; *n* = 5) at 20 s and 3 min. (**b**) Effect of the intravenous administration of *C. rhodostoma* venom (500–1000 µg/kg) on rat heart rate at 20 s and 3 min. * *p* < 0.05, compared to baseline (one-way ANOVA followed by Tukey’s multiple comparison test; *n* = 5).

*C. rhodostoma* venom (500–1000 µg/kg, i.v.) displayed hypotensive effects when administered to anaesthetized rats. *C. rhodostoma* venom (500 µg/kg, i.v., Figure 1b) decreased MAP to 45 ± 4 mmHg (Figure 2a) at 20 s after administration. While the higher dose of venom (i.e., 1000 µg/kg, i.v.) caused a similar reduction in MAP (43 ± 1 mmHg, Figure 2a) at 20 s, it also caused cardiovascular collapse (i.e., undetectable blood pressure) within 4 min (Figure 1c).

*C. rhodostoma* venom (500–1000 µg/kg, i.v.) also caused a significant reduction in heart rate at 20 s (Figure 2b), with an abolition of heart rate with the higher dose of venom after 3 min (Figure 2b).

**Figure 3:**
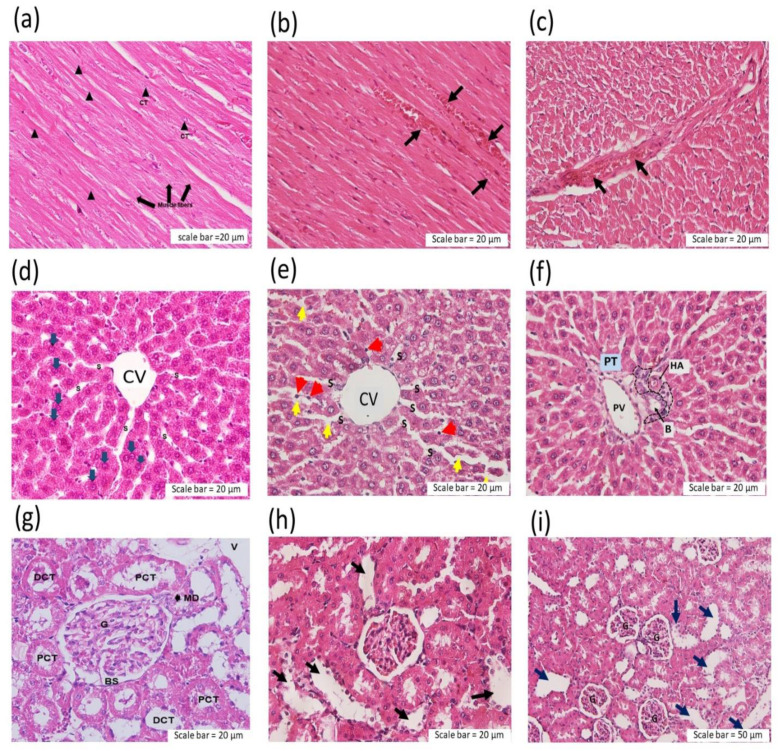
Histopathological examination of heart tissue (H&E staining) 6 h after the intravenous administration of (**a**) saline (200 µL: black triangles—intercalated discs; CT—centrally located nucleus) or *C. rhodostoma* venom at (**b**) 500 µg/kg (longitudinal section; arrows indicate the aggregation of red blood cells) or (**c**) 1000 µg/kg (transverse section; black arrows indicate the aggregation of red blood cells) in anaesthetized rats. Pathological changes in liver tissue 6 h after the intravenous administration of (**d**) saline (200 µL; S—sinusoid; blue arrow—hepatocyte; CV—central vein) or (**e**) *C. rhodostoma* venom (500 µg/kg), which caused sinusoid (s) dilatation (yellow arrows) and hepatocyte cell necrosis (red arrows). At the portal triad (PT), (**f**) the aggregation of polymorphonuclear leucocytes (PMNs) was detected following the administration of *C. rhodostoma* venom (500 µg/kg, i.v.; PV—portal vein; B—bile duct; HA—hepatic artery). Morphological changes in the kidney following the administration of (**g**) saline (200 µL; PCT—proximal convoluted tubule; DCT—distal convoluted tubule; G—glomerulus; MD—macula densa; BS—Bowman’s capsule). The administration of *C. rhodostoma* venom at a dose of (**h**) 500 µg/kg) and (**i**) 1000 µg/kg caused tubular dilatation (arrows indicate tubular dilatation).

At the conclusion of the experiment, all animals that were still alive were killed by an overdose of pentobarbitone after 6 h. The liver, heart and kidneys were removed for further histopathological studies. The administration of *C. rhodostoma* venom (500 and 1000 µg/kg, i.v.) caused thrombus formation in the cardiac myocardium (Figure 3b,c). In the liver tissue from envenomed animals, sinusoid (S) dilation and hepatocyte cell necrosis were detected at the central vein (CV) following experimental envenoming (Figure 3e). *C. rhodostoma* venom (500–1000 µg/kg, i.v.) also caused the aggregation of polymorphonuclear leucocyte (PMN) around the portal triad (PT), which contains the portal vein (PV), hepatic artery (HA) and bile duct (B) (Figure 3f).

Morphological changes were detected in the kidneys following the intravenous administration of *C. rhodostoma* venom as the presence of glomerular atrophy with renal tubule injury and dilatation (Figure 3h,i).

### 2.2. Neutralization of Venom Lethality In Vivo by Hemato Polyvalent Antivenom in Mice

The lethal effect (expressed as murine LD_50_) of *C. rhodostoma* venom was 3.706 mg/kg (i.p.). To determine the neutralizing effect of HPAV on *C. rhodostoma* venom lethality, we challenged groups of mice with three times the respective LD_50_ dose of venom (i.e., 11.1 mg/kg, i.p..; 3 × LD_50_) and determined the effectiveness of the administration of HPAV 15 min prior to and 15 min after the intraperitoneal administration of *C. rhodostoma* venom. The prior administration (i.e., 15 min) of HPAV at the recommended titre neutralised the toxicity of *C. rhodostoma* venom, whereas the administration of HPAV (i.p., recommended titre: 1 mL of antivenom to neutralise 1.6 mg of Malayan pit viper venom) 15 min after venom administration still resulted in the deaths of 25% of the animals tested.

### 2.3. Evaluation of the Effectiveness of Hemato Polyvalent Antivenom on C. rhodostoma Venom-Induced Morphological Changes in Mice

#### 2.3.1. Effect of *C. rhodostoma* Venom on Morphological Changes in the Liver and the Effect of HPAV on the Histopathology of the Liver

**Figure 4:**
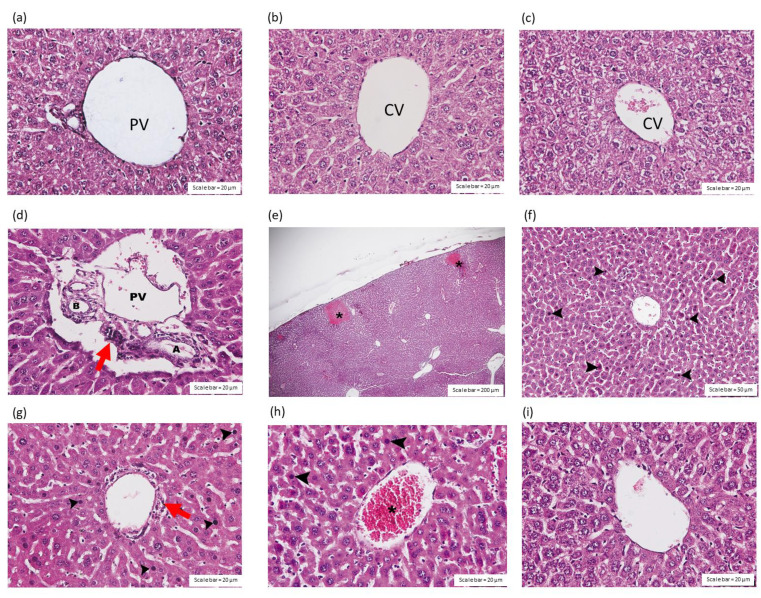
Histopathological changes (H&E staining) in (**a**) the portal triad (PV—portal vein) and (**b**) the central vein (CV) of the liver 24 h after the intraperitoneal administration of saline. (**c**) Effect of the administration of HPAV alone on the central vein of the liver. The intravenous administration of *C. rhodostoma* venom (3 × LD_50_), 24 h prior, induced the inflammation of hepatocytes at (**d**) the portal triad (scale bar = 20 µm; B—bile duct; PV—portal vein; A—hepatic artery) indicated by the presence of leukocyte infiltration (red arrow), (**e**) the presence of amyloidosis (asterisks; scale bar = 200 µm), (**f**) hepatic necrosis with pyknotic nuclei and eosinophilic cytoplasm (scale bar = 50 µm) and (**g**) polymorphonuclear leukocyte aggregation (red arrow; scale bar = 20 µm). The administration of *C. rhodostoma* venom (3 × LD_50_) also induced (**h**) congestion in the central vein, indicated by the presence of red blood cells (asterisk). (**i**) The protective effect of the prior administration (15 min) of HPAV on *C. rhodostoma* venom-induced hepatotoxicity (scale bar = 20 µm). Black arrowhead indicates pyknotic nuclei and eosinophilic cytoplasm.

The intraperitoneal administration of saline (Figure 4a,b) or HPAV (Figure 4c) did not affect the portal triad or central vein in the liver tissues. The administration of *C. rhodostoma* venom (3 × LD_50_; i.p.) caused inflammation in the hepatocytes around the portal triad (Figure 4d); congestion in the sinusoids; amyloidosis (Figure 4e); diffused hepatic necrosis, including oedema in hepatocyte cells (Figure 4f); and congestion in the central vein (Figure 4h). Inflammatory cells, including lymphocytes and Kupffer cells, were detected throughout the liver tissues from envenomed mice, including in the portal triad (Figure 4g). An absence of amyloidosis and a decrease in the vascular congestion of the central vein of the liver were detected (Figure 4i) in mice that received the recommended doses of HPAV either before or after the administration of *C.* *rhodostoma* venom (Table 1).

**Figure 5:**
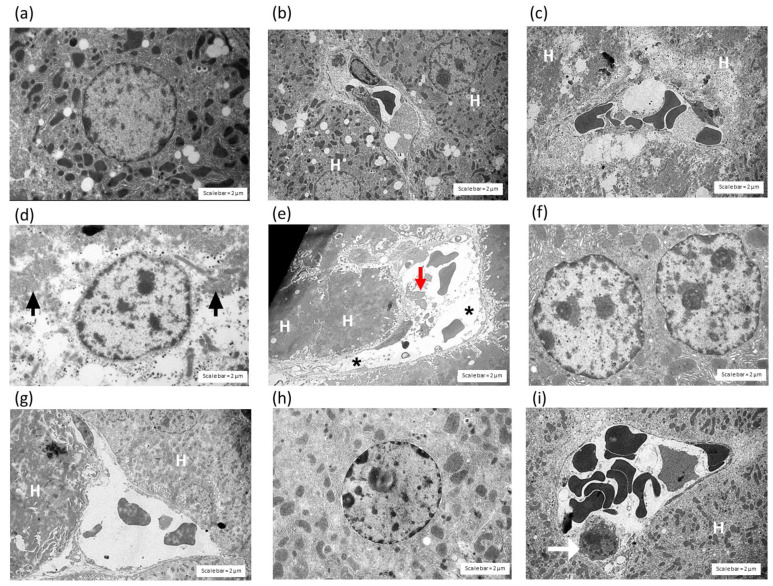
Histopathological examination of the liver tissue from mice using transmission electron microscopy (TEM). Effect of the administration of saline (i.p.) on (**a**) hepatocytes and (**b**) sinusoids. The administration of (**c**) HPAV did not cause any evidence of histopathological changes (H—hepatocyte). The administration of *C. rhodostoma* venom (3 × LD_50_, i.p.) caused (**d**) the disappearance of normal cell organization and swollen mitochondrial structures (black arrows) and (**e**) necrotic hepatocytes and the presence of cell debris in liver sinusoids (red arrow). Tissues from mice administered HPAV 15 min prior to *C. rhodostoma* venom showed a small degree of hepatotoxicity in the (**f**) hepatocytes and (**g**) sinusoids. Effect of the administration (i.p.) of HPAV 15 min after venom administration on the morphological changes in (**h**) hepatocytes and (**i**) sinusoids. The white arrow indicates a Kupffer cell). Scale bar = 2 µm. Asterisks indicate dilated blood sinusoid of hepatocytes.

The administration of saline (Figure 5a,b) and HPAV (Figure 5c) did not induce histopathological effects on the liver tissue that were detectable by TEM. In contrast, the administration of *C. rhodostoma* venom (3 × LD_50_; i.p.) caused the diffusion of necrotic hepatocytes and mitochondrial swelling (Figure 5d), together with damage in the cytoplasm. These effects were indicated by the presence of dilated blood sinusoids around the boundary of the hepatocytes. Expanded sinusoids with cell debris were also observed in the livers of envenomed mice (Figure 5e). Moreover, the administration of HPAV 15 min prior to or after the administration of *C. rhodostoma* venom minimized the level of lymphocytes (Figure 5g,i) as well as the pyknotic nuclei and eosinophilic cytoplasm in the necrotic hepatocytes (Figure 5f,h).

#### 2.3.2. Effect of *C. rhodostoma* Venom on Morphological Changes in Heart Tissues

**Figure 6:**
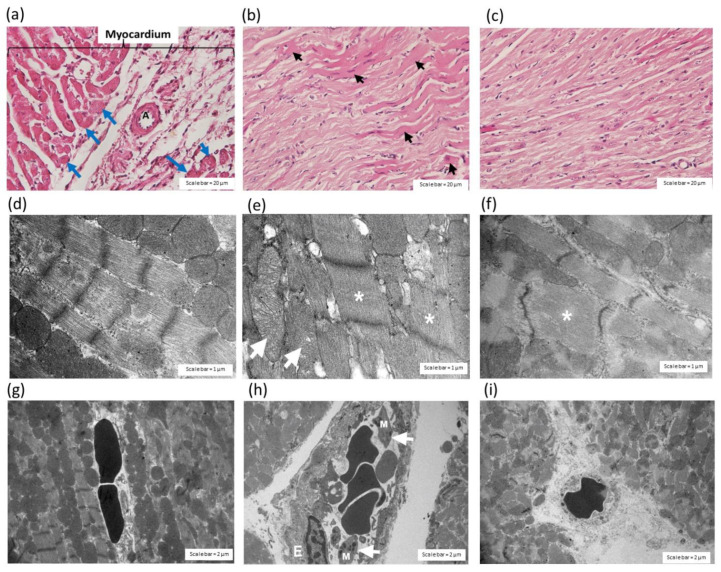
Histopathological examination of heart tissue (H&E staining) 24 h after intraperitoneal administration of *C.* *rhodostoma* venom (3 × LD_50_) in mice causing (**a**) an irregular shape of muscle fibres in the myocardium (blue arrows) and (**b**) the hypertrophy of cardiac muscle fibres (black arrows; scale bar = 20 µm). (**c**) Protective effect of the administration of HPAV on *C. rhodostoma* venom-induced cardiac muscle fibre damage prior to venom exposure (scale bar = 20 µm). (**d**) Histopathological changes in cardiac muscle fibres following the administration of saline under TEM. The administration of *C. rhodostoma* venom (3 × LD_50_, i.p.) caused (**e**) cardiac muscle fibre hypertrophy and mitochondrial swelling (white arrows; scale bar = 1 µm). (**f**) Protective effect of the administration of HPAV prior to venom exposure against cardiac muscle hypertrophy in *C. rhodostoma* venom-induced cardiac muscle fibre damage. (**g**) TEM image of cardiac vessels following the administration of saline. The histopathological investigation of heart tissue under TEM indicated that the administration of *C. rhodostoma* venom (3 × LD_50_, i.p.) caused (**h**) endothelial cell swelling (E; scale bar = 2 µm) and an increase in the presence of macrophages (white arrows). (**i**) The protective effect of the prior administration (15 min) of HPAV on *C. rhodostoma* venom-induced cardiac tissue damage (white asterisk indicates cardiac muscle fibre hypertrophy and M indicates macrophage infiltration).

The administration of *C. rhodostoma* venom, 24 h prior, induced irregularly shaped muscle fibres in the myocardium (Figure 6a) and the hypertrophy of cardiac muscle fibres (Figure 6b). The administration of HPAV, prior to the administration of *C. rhodostoma* venom, prevented endothelial cell swelling (Figure 6c). The administration of saline (i.p.) caused no apparent histopathological changes in the heart tissue (Figure 6d,g). After the administration of *C. rhodostoma* venom (3 × LD_50_), 24 h prior, cardiac muscle fibre hypertrophy and mitochondrial swelling were found (Figure 6e). Cardiac vessels also showed endothelial cell swelling and macrophage infiltration (Figure 6h) following the intraperitoneal administration of *C. rhodostoma* venom (3 × LD_50_). Venom-induced pathological changes in cardiac muscle fibres were minimized by the administration of HPAV 15 min prior to or after to the administration of *C. rhodostoma* venom (Figure 6f,i; Table 2).

#### 2.3.3. Effect of *C. rhodostoma* Venom on Morphological Changes in the Kidneys and the Protective Effect of HPAV on the Pathohistology of the Kidneys

**Figure 7:**
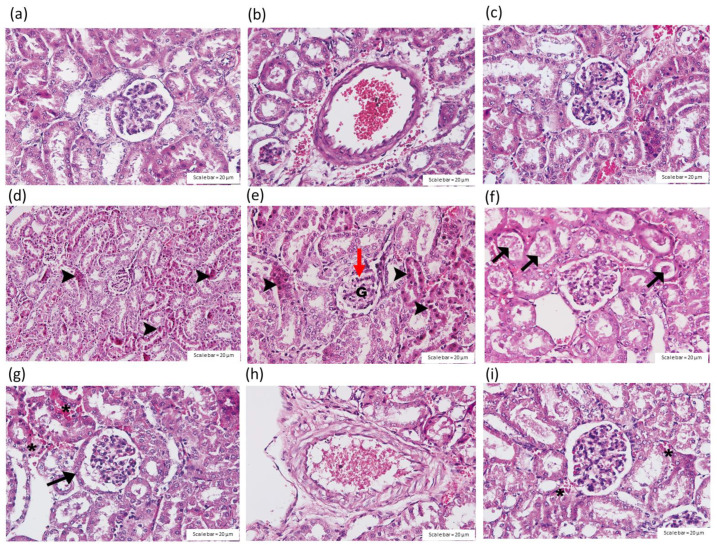
Histopathological changes in the kidney tissues of mice (H&E staining; scale bar = 20 µm) 24 h after intraperitoneal administration of saline in the (**a**) glomerulus and (**b**) interlobar artery. (**c**) Effect of the intraperitoneal administration of HPAV on the glomerulus. The intraperitoneal administration of *C. rhodostoma* venom (3 × LD_50_) caused (**d**) tubular necrosis, (**e**) glomerular atrophy (red arrow), (**f**) tubular cast (black arrow), (**g**) interstitial vessel congestion in the glomerulus (asterisks) and the hypertrophy of parietal cells (black arrow) and (**h**) the disintegration of the tunica intima of the arterial wall. (**i**) A small degree of interstitial vessel congestion was observed following the administration of HPAV prior to venom administration. * interstitial vessel congestion; ►tubular necrosis.

**Figure 8:**
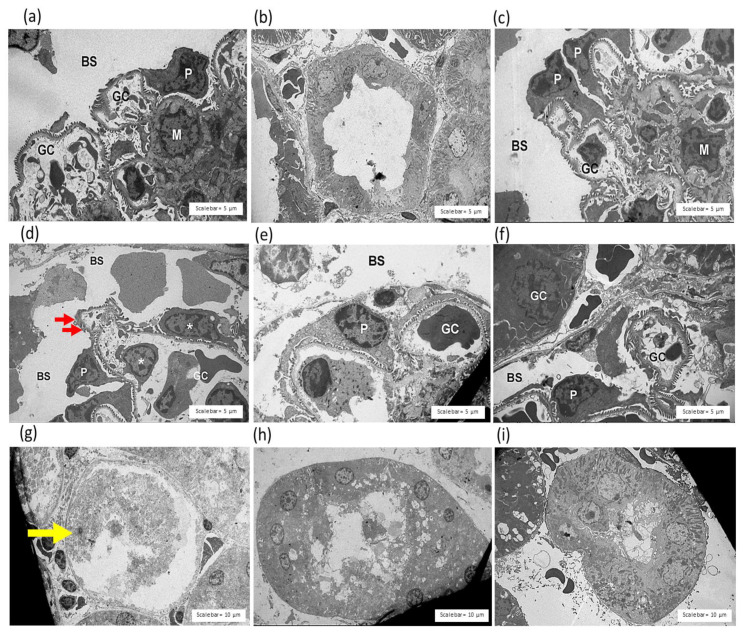
The histopathological examination of mouse kidneys under TEM indicating the effect of the intraperitoneal administration of saline on the (**a**) glomerulus and (**b**) renal tubule. (**c**) Effect of intraperitoneal HPAV on the glomerulus. *C. rhodostoma* venom (3 × LD_50_, i.p.) caused (**d**) tubular injury in the glomerulus, the disarrangement of pedicels (red arrow) and endothelial cell swelling (white asterisks). The histopathological study showed (**e**) dilated glomerular capillaries in the mice that received HPAV 15 min after the administration of *C. rhodostoma* venom. (**f**) Protective effect of the prior administration of HPAV on venom-induced morphological changes in the glomerulus. The administration of *C. rhodostoma* venom (3 × LD_50_, i.p.) in mice caused (**g**) a loss of cellular organelles in the cytoplasm of the renal tubules (yellow arrow). The histopathological study showed (**h**) tubular cell swelling in the renal tubules of the mice that received HPAV 15 min after the administration of *C. rhodostoma* venom. (**i**) Effect of the prior administration of HPAV on venom-induced morphological changes in the renal tubule. BS—Bowman’s space; GC—glomerular capillaries; P—podocyte; M—mesangium cell.

The administration of saline and HPAV did not cause histopathological changes in the kidneys that were observable under a light microscope (Figure 7a–c) or TEM (Figure 8a–c).

The histopathological analysis of mouse kidneys 24 h after exposure to crude *C. rhodostoma* venom (3 × LD_50_; i.p.) indicated tubular injury with a loss of the brush border (Figure 7d), the dilatation of renal capillaries, diffuse or focal glomerular atrophy (Figure 7e), hyaline cast (Figure 7f) and/or the congestion of interstitial vessels (Figure 7g). The disarrangement of kidney vascular smooth muscle was also detected in envenomed tissues (Figure 7h).

The histopathological study of mouse kidneys under TEM revealed the disarrangement of pedicels and glomerular rupture (Figure 8d). Tubular injury and the lysis of tubular nuclei were detected in the kidneys from envenomed mice (Figure 8g).

Dilated glomerular capillaries (Figure 8e) and the swelling of renal tubule cells (Figure 8h) were rarely detected in the mice that received HPAV 15 min after the administration of *C. rhodostoma* venom. The administration of HPAV 15 min prior to or after venom administration decreased tubular cast, interstitial vessel congestion (Figure 7i) and vacuolar degeneration (Figure 8f,i; Table 3).

## 3. Discussion

*Calloselasma rhodostoma* is a pit viper species endemic to Southeast Asia. Severe local effects, including swelling, blistering, compartment syndrome and tissue necrosis, are commonly observed following envenoming by *C. rhodostoma* in Thailand [16]. In addition, coagulopathy resulting in haemorrhage is a major systemic outcome in *C. rhodostoma* envenomed patients [15]. *C. rhodostoma* envenoming-induced acute kidney injury and cardiovascular events, i.e., congestive heart failure, were also reported in a patient who had a long length of hospital stay in southern Thailand [15]. This prompted us to investigate the pathological effects behind nephrotoxicity and cardiovascular disturbances using histopathological analysis. We also examined the effectiveness of HPAV in inhibiting morphological changes following *C. rhodostoma* envenoming. In this study, anaesthetized rats were used to examine cardiovascular effects following the intravenous administration of Malayan pit viper venom. The venom induced cardiac collapse within 3–5 min, suggesting a direct effect of the venom on vasculature and/or cardiac function. In addition, we determined the median murine lethal dose (LD_50_) for Malayan pit viper venom using the WHO-recommended protocol [20].

The administration of monospecific antivenom remains an effective treatment for viper envenomings. However, the availability of, and access to, geographically appropriate antivenom and the correct identification of the biting species remain problematic in many rural areas. The administration of polyvalent antivenom is a valid option for snakebite patients in order to minimize the occurrence of incorrect antivenom application due to diagnostic error. A number of studies have exhibited the effectiveness of Hemato polyvalent antivenom (HPAV) from Thailand in inhibiting the toxicity of various Asian haematotoxic and nephrotoxic snakes, i.e., *Daboia* spp., *Trimeresurus* spp. and *Challoselasma* spp., including *Hypnale* spp. [17,18,19]. HPAV was demonstrated to effectively neutralise the procoagulant and haemorrhagic activities of all venoms tested. Moreover, HPAV also displayed a preventive effect against the occurrence of *Daboia siamensis* venom-induced haematuria and proteinuria in envenomed animals [18]. Interestingly, the potency of HPAV was shown to be generally higher than that of *C. rhodostoma* monovalent antivenom in the neutralization of lethality, coagulation, haemorrhage and necrosis associated with a challenge dose (5 × LD_50_) of *C. rhodostoma* venom, suggesting the presence of a higher level of antibodies and a synergistic cross-neutralizing component of HPAV [18]. Similarly, a previous study also indicated that the early administration of *Daboia siamensis* monovalent antivenom at concentrations higher than the recommended titre (i.e., 1 mL of antivenom for 0.6 mg of *Daboia siamensis* venom) was required to prevent nephrotoxicity following Russell’s viper envenoming [19]. In the present study, the administration of HPAV at the recommended concentration (1 mL of antivenom to neutralise 1.6 mg of Malayan pit viper venom) displayed neutralizing activity against histopathological changes in the heart, liver and kidney tissues when given either 15 min prior to or 15 min after the administration of *C. rhodostoma* venom (3 × LD_50_) in experimentally envenomed mice. In a preliminary study, we found that the intraperitoneal administration of *C. rhodostoma* venom (3 × LD_50_) was lethal in animals tested within 1 h (45 ± 28 min; *n* = 5). Therefore, the administration of HPAV 15 min after *C. rhodostoma* envenoming was chosen as a suitable time point to examine the effectiveness of HPAV after envenoming. There were no remarkable differences in the protective effects of HPAV in envenomed mice when the antivenom was administered either 15 min prior to or after envenoming.

*C. rhodostoma* venom is a rich source of biological proteins such as snake venom metalloproteinase (SVMPs), phosphodiesterase (PDEs), phospholipase A_2_ (PLA_2_) and snake venom serine proteases (SVSPs). Recently, aminopeptidase, glutaminyl-peptide cyclotransferase and ankyrin repeats were identified in Malaysian *C. rhodostoma* venom [8]. These toxic proteins have been shown to be responsible for a number of hematologic outcomes (e.g., haemorrhage, hypotension and inflammation) [21,22] and cellular necrosis [23,24], which may involve the nephrotoxicity, hepatotoxicity and cardiovascular disturbances observed following *C. rhodostoma* envenoming.

Cardiovascular effects following snakebite envenoming have been reported in victims of snakes from the Elapidae (i.e., *Pseudonaja textilis*, *Oxyuranus scutellatus* and *Bungarus candidus*) and Viperidae (i.e., *Echis ocellatus*) families. In this study, the intravenous administration of 500 µg/kg of *C. rhodostoma* venom caused a rapid but transient decrease in blood pressure and heart rate, followed by a more prolonged hypotensive effect for a few minutes. In contrast, while the administration of 1000 µg/kg of *C. rhodostoma* venom also lowered mean arterial pressure and heart rate, this was followed by cardiovascular collapse. However, the intraperitoneal administration of Malayan pit viper venom in mice (3 × LD_50_) did not cause the sudden collapse seen in anaesthetized rats. The different routes of administration and, hence, the different times for key venom toxins to reach their site of action (i.e., slower under i.p. administration), are likely to be responsible for these different outcomes. The mechanism behind the transient hypotension and cardiovascular collapse following snakebite envenoming has been demonstrated to involve vascular mediators (e.g., nitric oxide and prostacyclin) and autonomic adaptation [25,26]. Previously, we reported that OSC3, a PLA_2_ isolated from *O. scutellatus* (Taipan) venom, induced a transient decrease in MAP and vascular relaxation in the mesenteric arteries of anaesthetized rats, which was due to a combination of the release of dilator autacoids and the direct relaxation of vascular smooth muscle involving the cAMP/protein kinase A cascade [26,27]. In addition, we showed that the prior administration of hexamethonium or atropine significantly attenuated the cardiac toxicity of Malayan krait (*B. candidus*) venom [25], suggesting the involvement of ganglionic nicotinic receptors and muscarinic acetylcholine receptors. Moreover, the administration of a prothrombin activator from *P. textilis* venom induced cardiovascular collapse in anaesthetized rats, suggesting that prothrombin activator-like toxin may be a contributor to snake venom-induced rapid cardiovascular collapse [28]. Indeed, acute coronary syndrome was reported in a *C. rhodostoma* envenomed patient and was relieved by the administration of antithrombotic agents for 5 days [16]. In our current study, *C. rhodostoma* venom caused swelling in the vasculature of cardiac muscle and was associated with the presence of macrophages in cardiac vessels, indicating a direct effect of the venom on tissues. The histopathological examination of heart tissue indicated that *C. rhodostoma* venom caused extensive hypertrophy of cardiac myofibers and mitochondrial swelling after envenoming. These morphological changes in cardiac tissues can be attributed to the presence of cellular cytotoxic components in venom.

In this work, the effect of *C. rhodostoma* venom on the histopathology of liver tissue was also investigated. Hepatocyte vacuolation, prominent van Kupffer cells and congestion in the central vein were detected in the liver. We also detected the presence of lymphocytes, pyknotic nuclei and eosinophilic cytoplasm, causing amyloidosis in some areas. This indicated the presence of an inflammatory effect on the hepatic tissue. In fact, these hepatic injuries were also observed following *Naja haje* [29] and *Crotalus durissus terrificus* [30] envenoming. The hepatotoxic effects included an elevation in bilirubin and increases in serum alanine, aminotransferase, aspartate aminotransferase, γ-glutamyl transferase and alkaline phosphatase. The mechanism behind snake venom-induced hepatotoxicity was previously demonstrated to be associated with liver apoptosis, as indicated by a rise in lipid peroxidation and nitric oxide production [29]. In fact, snake venom L-amino acid oxidases (LAAOs) have become important cytotoxic agents, causing cell death in several organisms via the release of reactive oxygen species, including hydrogen peroxide (H_2_O_2_) [31].

There are 5 groups of Asian snakes that have been reported to cause nephrotoxicity, i.e., Russell’s vipers, green pit vipers, saw-scaled vipers, hump-nosed pit vipers and sea-snakes. In the present work, we demonstrated the nephrotoxicity induced by *C. rhodostoma* venom. In Thailand, acute kidney injury and rhabdomyolysis (two patients) were clinically reported following *C. rhodostoma* envenoming. Of these patients, one died from rhabdomyolysis following recovery from systemic bleeding [16]. Nephrotoxicity is commonly induced by snakes with hemotoxic and myotoxic effects, e.g., vipers, Australian elapids and sea snakes. The clinical manifestations of renal involvement include proteinuria, haematuria, pigmenturia and acute kidney injury [32]. We previously demonstrated that Asian Russell’s viper (*Daboia* spp.) venom contains nephrotoxic substances, e.g., SVPLA_2_ and SVMP, which cause glomerulonephritis, interstitial congestion, tubular necrosis and cortical necrosis in envenomed tissues [33]. The present study showed that *C. rhodostoma* venom caused tubular necrosis with cytoplasmic eosinophilia and pyknotic nuclei, indicating the presence of inflammation in the renal tubule, similar to the nephrotoxic lesions induced by other species. To treat and prevent nephrotoxicity, apart from the administration of snake antivenom, early plasmapheresis and blood exchange have been applied when snake antivenom is unavailable. Moreover, plasmapheresis, blood exchange, and peritoneal or haemodialysis should be performed as early as possible for the prevention of AKI. In addition, urine alkalization by sodium bicarbonate may also help to prevent AKI in patients with myoglobinuria or haemoglobinuria [32].

## 4. Conclusions

In this study, we demonstrated that *C. rhodostoma* envenoming causes profound histopathological changes in the heart, liver and kidney tissues. Further pharmacological and physiological determinations may enable a better understanding and management of Malayan pit viper envenoming. Our data also indicate that the morphological anomalies observed in envenomed tissues may contribute to cardiovascular disturbances and nephrotoxicity in envenomed victims. The early monitoring of cardiovascular and renal function together with appropriate snake antivenom administration are required to prevent life-threatening outcomes.

## 5. Materials and Methods

### 5.1. Snake Venoms

Malayan pit viper venom (*C. rhodostoma* venom, batch number 607.002), pooled and lyophilized from 9 Indonesian specimens and 62 Malaysian specimens, was purchased from Latoxan (Valence, France). Freeze-dried venom samples were stored at 4 °C prior to use. When required, the venom was weighed and reconstituted in phosphate-buffered saline (PBS), and the protein concentration was measured using a BCA protein assay (Pierce Biotechnology, Rockford, IL, USA).

### 5.2. Antivenoms

Hemato polyvalent snake antivenom (HPAV; Lot No. HP00216; expiry date 4 August 2022) was purchased from the QSMI of the Thai Red Cross Society, Bangkok, Thailand. The freeze-dried antivenoms were dissolved with pharmaceutical-grade water supplied by the manufacturer. The dissolved antivenoms were then stored at 4 °C prior to use.

### 5.3. Animal Ethics and Care

All methods were performed in accordance with the relevant guidelines and regulations (https://arriveguidelines.org accessed on 20 July 2021). In brief, male Sprague–Dawley rats and Jcl-ICR mice were purchased from Nomura-Siam International Co., Ltd., Bangkok, Thailand. All animals were maintained on a regular diurnal lighting cycle (12:12 light:dark). Two or four rats were housed together in individual stainless-steel cages with access to food and drinking water ad libitum. Chopped corn cob was used as bedding. Approvals for all experimental procedures were obtained from the Subcommittee for Multidisciplinary Laboratory and Animal Usage of Phramongkutklao College of Medicine and the Institutional Review Board, Royal Thai Army Department, Bangkok, Thailand (documentary proof of ethical clearance number: IRBRTA S055b/64_Xmp and IRBRTA S054b/64_Xmp on 19 July 2021) in accordance with the 1986 U.K. Animal (Scientific Procedure) Act and the National Institutes of Health’s *Guide for the Care and Use of Laboratory Animals* (NIH Publications 8th edition, 2011).

### 5.4. Anaesthetized Rat Preparation

Male Sprague–Dawley rats weighting 280–350 g (*n* = 4–5 per group) were anaesthetized using separate injections of Zoletil (20 mg/kg, i.p.) and Xylazine (5 mg/kg, i.p.). Additional anaesthetic was administered throughout the experiment as required. A midline incision was made in the cervical region, and cannulae were inserted into the trachea, jugular vein and carotid artery for artificial respiration (if required), the administration of drugs/venom and the measurement of blood pressure, respectively. Arterial blood pressure was recorded using a Gould Statham P23 pressure transducer filled with heparinized saline (25 U/mL). Systemic blood pressure was monitored with a MacLab system (ADInstruments). Pulse pressure was defined as the difference between systolic and diastolic blood pressures. Mean arterial pressure (MAP) was defined as diastolic blood pressure plus one-third of pulse pressure. The rats were kept under a heat lamp for the entire experiment to maintain body temperature.

### 5.5. In Vivo Venom Lethality

As an essential prerequisite to assessing antivenom efficacy, we first determined the median murine lethal dose (LD_50_) for *C. rhodostoma* venom using the WHO-recommended protocols [20]. Briefly, groups of 4–5 male Jcl:ICR mice (18–22 g) received an intraperitoneal injection of varying doses of venom in 100 µL PBS; 24 h later, the number of surviving mice in each group was recorded. The venom LD_50_ (i.e., the amount of venom that caused lethality in 50% of a population of injected mice) and the corresponding 95% confidence limits of each venom concentration were calculated using probit analysis [19,34].

To determine the effectiveness of HPAV, a challenge dose (3 × LD_50_) of *C. rhodostoma* venom was intraperitoneally administered to the animals. The neutralizing effect of HPAV (manufacturer recommended titre: 1 mL of antivenom to neutralise 1.6 mg of Malayan pit viper venom) was examined when it was administered 15 min prior to or 15 min after *C. rhodostoma* venom.

### 5.6. Histopathological Studies

#### 5.6.1. Histological Preparation for Haematoxylin and Eosin (H&E) Staining

The histopathological examination of the heart, liver and kidneys of envenomed animals was carried out following previously described methods [35]. At the conclusion of the in vivo tests (i.e., 24 h), the surviving mice and rats were sacrificed, and their liver, heart and both kidneys were removed and washed with phosphate-buffered saline and preserved in 10% formalin. All tissues were dehydrated in a graded series of ethanol through 70, 80, 90, 95 and 100% with two changes for 1 h each. Three washings of xylene for 30 min each were then completed before embedding the tissues in paraffin. The embedded samples were cut into cross-sections and stained with H&E. The tissues were examined and photographed using an Olympus light microscope (BX-50, Olympus, Tokyo, Japan). The degrees of severity of the morphological changes in the liver, heart and kidneys were evaluated as previously described [36,37] (Supplementary Material).

#### 5.6.2. Histological Preparation for Transmission Electron Microscopy

Pieces of liver, heart and kidney tissues (~1 mm^3^) were immediately fixed in 2.5% buffered glutaraldehyde. The specimens were post-fixed in 1% osmium tetroxide, dehydrated, infiltrated with propylene oxide and embedded in resin. Semi-thin sections of ~0.5 µm or 1.0 µm were stained with Toluidine blue, used as a guideline to the area of interest and further trimmed. Ultrathin sections of ~60 nm were cut on an ultramicrotome; then stained with uranyl acetate and lead citrate. The ultrathin sections were spread mostly on 200 or 300 mesh copper grids and stained with uranyl acetate and lead citrate solutions. The sections were then examined and photographed using a transmission electron microscope (TEM-JEM2010, JEOL, Ltd., Tokyo, Japan).

### 5.7. Data Analysis and Statistics

For the anaesthetized rat experiments, the sample sizes were based on the number of animals required to provide >85% power to detect an effect size of 35% with a confidence level (α) of 5% for the in vivo endpoint measure of blood pressure (standard deviation (SD) <15%). This ensured that the experimental design was sufficiently powered. The statistical analysis was performed using Prism 5.0 software (GraphPad Software, San Diego, CA, USA). Multiple comparisons were made using a one-way analysis of variance (ANOVA) followed by Tukey’s multiple comparison test. Data were expressed as mean ± SD.

## Figures and Tables

**Table 1:** Pathological score of liver tissues following the intraperitoneal administration of saline (negative control), *C. rhodostoma* venom or Hemato polyvalent antivenom 15 min prior to or after the administration of *C. rhodostoma* venom (0—undetectable lesion; +—pathological lesion). The degree of morphological changes in the liver is shown in Supplementary Material.

Group	Scores		
	Congestion	Inflammatory Infiltration	Necrosis
Saline	0	0	0
*C. rhodostoma* venom (CRV, i.p.)	+++	++	+++
HPAV (i.p.)	0	0	0
CRV followed by HPAV 15 min later	+	+	+
HPAV followed by CRV 15 min later	+	+	+

Congestion scores: 0—no congestion; +—in few sinusoids and vessels; +++—in almost all sinusoids and vessels. Inflammatory infiltration scores: 0—no inflammatory infiltrate; +—1-3 inflammatory foci/section; ++—4–6 inflammatory foci/section. Necrosis scores: 0—no necrosis; +—focal necrosis; +++—confluent necrosis.

**Table 2:** Pathological changes in the heart tissue following the intraperitoneal administration of saline (negative control), *C. rhodostoma* venom or Hemato polyvalent antivenom 15 min prior to or after the administration of *C. rhodostoma* venom (0—undetectable lesion; +—pathological lesion). The degree of morphological changes in cardiac muscle fibres is shown in Supplementary Material.

Group	Heart (Scores)	Description
	Myocardial Damage	
Saline	0	No lesions
*C. rhodostoma* venom (CRV, 11.1 mg/kg; i.p.)	2.5	Focal lesions extending over a wider area of both ventricles, extensive inflammatory cell infiltration, interstitial oedema, rupture of myofibers
HPAV (i.p.)	0.5	Slight derangement of muscle fibres, few inflammatory cells and vacuoles
CRV followed by HPAV	1.5	Focal lesions of the subendocardium of the apical and mid ventricular region with right ventricular involvement
HPAV followed by CRV	1.5	Focal lesions of the subendocardium of the apical and mid ventricular region with right ventricular involvement

**Table 3:** Pathological changes in kidney tissues following the intraperitoneal administration of saline (negative control), *C. rhodostoma* venom or Hemato polyvalent antivenom 15 min prior to or after the administration of *C. rhodostoma* venom (0—undetectable lesion; +—pathological lesion). The degree of morphological change in the kidneys is shown in Supplementary Material.

Group	Kidney (Scores)		
	Congestion	Inflammatory Infiltration	Tubular Injury
Saline	0	0	0
*C. rhodostoma* venom (CRV, i.p.)	+++	++	+++
HPAV (i.p.)	0	0	0
CRV followed by HPAV	+	+	+
HPAV followed by CRV	+	+	+

Kidney congestion scores: 0—no congestion; +—focal glomeruli and interstitial vessels; +++—in almost all glomeruli and interstitial vessels or haemorrhage. Kidney inflammatory infiltration scores: 0—no inflammatory infiltrate; +—1-3 inflammatory foci/section; ++—4-6 inflammatory foci/section. Tubular injury scores: 0—no evidence of tubular injury; +—less than 50% loss of brush border; +++—tubular necrosis found in most areas.

## Data Availability

The datasets used and/or analysed during the current study are available from the corresponding author (J.C.) upon reasonable request.

## Supplementary Materials

The following are available online at www.mdpi.com/article/10.3390/toxins14090601/s1, Table S1: Pathological characteristic and evaluation criteria for degree of morphological changes in liver, Table S2: Pathological characteristic and evaluation criteria for degree of morphological changes in kidneys, Table S3: Pathological characteristic and evaluation criteria for degree of morphological changes in heart.

## References

[1] Longbottom J., Shearer F.M., Devine M., Alcoba G., Chappuis F., Weiss D.J., Ray S.E., Ray N., Warrell D.A., Ruiz de Castaneda R. (2018). Vulnerability to snakebite envenoming: A global mapping of hotspots. Lancet.

[2] Gutierrez J.M., Calvete J.J., Habib A.G., Harrison R.A., Williams D.J., Warrell D.A. (2017). Snakebite envenoming. Nat. Rev. Dis. Primers.

[3] WHO (2016). Venomous snakes of the South-East Asia Region, their venoms and pathophysiology of human envenoming. Guidelines for the Management of Snake-Bites.

[4] Ismail A.K., Gopalakrishnakone P. (2015). Snakebite and Envenomation Management in Malaysia. Clinical Toxinology in Asia Pacific and Africa, Toxinology.

[5] Tang E.L., Tan C.H., Fung S.Y., Tan N.H. (2016). Venomics of *Calloselasma rhodostoma*, the Malayan pit viper: A complex toxin arsenal unraveled. J. Proteom..

[6] Tang E.L.H., Tan N.H., Fung S.Y., Tan C.H. (2019). Comparative proteomes, immunoreactivities and neutralization of procoagulant activities of *Calloselasma rhodostoma* (Malayan pit viper) venoms from four regions in Southeast Asia. Toxicon.

[7] Bruserud O. (2013). The snake venom rhodocytin from *Calloselasma rhodostoma*- a clinically important toxin and a useful experimental tool for studies of C-type lectin-like receptor 2 (CLEC-2). Toxins.

[8] Kunalan S., Othman I., Syed Hassan S., Hodgson W.C. (2018). Proteomic Characterization of Two Medically Important Malaysian Snake Venoms, *Calloselasma rhodostoma* (Malayan Pit Viper) and *Ophiophagus hannah* (King Cobra). Toxins.

[9] Wongtongkam N., Wilde H., Sitthi-Amorn C., Ratanabanangkoon K. (2005). A study of 225 Malayan pit viper bites in Thailand. Mil. Med..

[10] Gutierrez J.M., Ponce-Soto L.A., Marangoni S., Lomonte B. (2008). Systemic and local myotoxicity induced by snake venom group II phospholipases A_2_: Comparison between crotoxin, crotoxin B and a Lys49 PLA2 homologue. Toxicon.

[11] Gutierrez J.M., Ownby C.L. (2003). Skeletal muscle degeneration induced by venom phospholipases A_2_: Insights into the mechanisms of local and systemic myotoxicity. Toxicon.

[12] Gutierrez J.M., Rucavado A., Chaves F., Diaz C., Escalante T. (2009). Experimental pathology of local tissue damage induced by *Bothrops asper* snake venom. Toxicon.

[13] Sitprija V. (2006). Snakebite nephropathy. Nephrology (Carlton).

[14] Kasempimolporn S., Jitapunkul S., Sitprija V. (2008). Moving towards the elimination of rabies in Thailand. J. Med Assoc. Thail. = Chotmaihet Thangphaet.

[15] Kraisawat K., Promwang N. (2020). Duration after Malayan Pit Viper Bite to Detect Coagulopathy in Songklanagarind Hospital. J. Health Sci. Med. Res..

[16] Tangtrongchitr T., Thumtecho S., Janprasert J., Sanprasert K., Tongpoo A., Tanpudsa Y., Trakulsrichai S., Wananukul W., Srisuma S. (2021). Malayan Pit Viper Envenomation and Treatment in Thailand. Ther. Clin. Risk Manag..

[17] Chaisakul J., Rusmili M.R.A., Alsolaiss J., Albulescu L.O., Harrison R.A., Othman I., Casewell N.R. (2020). In Vitro Immunological Cross-Reactivity of Thai Polyvalent and Monovalent Antivenoms with Asian Viper Venoms. Toxins.

[18] Leong P.K., Tan C.H., Sim S.M., Fung S.Y., Sumana K., Sitprija V., Tan N.H. (2014). Cross neutralization of common Southeast Asian viperid venoms by a Thai polyvalent snake antivenom (Hemato Polyvalent Snake Antivenom). Acta Trop..

[19] Chaisakul J., Alsolaiss J., Charoenpitakchai M., Wiwatwarayos K., Sookprasert N., Harrison R.A., Chaiyabutr N., Chanhome L., Tan C.H., Casewell N.R. (2019). Evaluation of the geographical utility of Eastern Russell’s viper (*Daboia siamensis*) antivenom from Thailand and an assessment of its protective effects against venom-induced nephrotoxicity. PLoS Negl. Trop. Dis..

[20] WHO (2010). Guidelines for the Production, Control and Regulation of Snake Antivenom Immunoglobulins. Geneva. https://www.who.int/biologicals/expert_committee/Antivenom_WHO_Guidelines_DJW_DEB_mn_cp.pdf.

[21] Markland F.S., Swenson S. (2013). Snake venom metalloproteinases. Toxicon.

[22] Chippaux J.P. (2006). Venomous and poisonous animals. II. Viper bites. Med. Trop. Rev. Du Corps De Sante Colonial.

[23] Uzair B., Atlas N., Malik S.B., Jamil N., Ojuolape S.T., Rehman M.U., Khan B.A. (2018). Snake Venom as an Effective Tool against Colorectal Cancer. Protein Pept. Lett..

[24] Kini R.M. (2003). Excitement ahead: Structure, function and mechanism of snake venom phospholipase A_2_ enzymes. Toxicon.

[25] Chaisakul J., Rusmili M.R., Hodgson W.C., Hatthachote P., Suwan K., Inchan A., Chanhome L., Othman I., Chootip K. (2017). A Pharmacological Examination of the Cardiovascular Effects of Malayan Krait (*Bungarus candidus*) Venoms. Toxins.

[26] Chaisakul J., Isbister G.K., Konstantakopoulos N., Tare M., Parkington H.C., Hodgson W.C. (2012). In vivo and in vitro cardiovascular effects of Papuan taipan (*Oxyuranus scutellatus*) venom: Exploring “sudden collapse”. Toxicol. Lett..

[27] Chaisakul J., Isbister G.K., Tare M., Parkington H.C., Hodgson W.C. (2014). Hypotensive and vascular relaxant effects of phospholipase A_2_ toxins from Papuan taipan (*Oxyuranus scutellatus*) venom. Eur. J. Pharmacol..

[28] Chaisakul J., Isbister G.K., O’Leary M.A., Parkington H.C., Smith A.I., Hodgson W.C., Kuruppu S. (2015). Prothrombin activator-like toxin appears to mediate cardiovascular collapse following envenoming by *Pseudonaja textilis*. Toxicon.

[29] Al-Quraishy S., Dkhil M.A., Abdel Moneim A.E. (2014). Hepatotoxicity and oxidative stress induced by *Naja haje* crude venom. J. Venom. Anim. Toxins Incl. Trop. Dis..

[30] Barraviera B., Coelho K.Y., Curi P.R., Meira D.A. (1995). Liver dysfunction in patients bitten by *Crotalus durissus terrificus* (Laurenti, 1768) snakes in Botucatu (State of Sao Paulo, Brazil). Rev. Do Inst. De Med. Trop. De Sao Paulo.

[31] Naumann G.B., Silva L.F., Silva L., Faria G., Richardson M., Evangelista K., Kohlhoff M., Gontijo C.M., Navdaev A., de Rezende F.F. (2011). Cytotoxicity and inhibition of platelet aggregation caused by an l-amino acid oxidase from *Bothrops leucurus* venom. Biochim. Et Biophys. Acta.

[32] Kanjanabuch T., Sitprija V. (2008). Snakebite nephrotoxicity in Asia. Semin. Nephrol..

[33] Chaisakul J., Khow O., Wiwatwarayos K., Rusmili M.R.A., Prasert W., Othman I., Abidin S.A.Z., Charoenpitakchai M., Hodgson W.C., Chanhome L. (2021). A Biochemical and Pharmacological Characterization of Phospholipase A_2_ and Metalloproteinase Fractions from Eastern Russell’s Viper (*Daboia siamensis*) Venom: Two Major Components Associated with Acute Kidney Injury. Toxins.

[34] Finney D. (1971). Probit Analysis.

[35] Charoenpitakchai M., Wiwatwarayos K., Jaisupa N., Rusmili M.R.A., Mangmool S., Hodgson W.C., Ruangpratheep C., Chanhome L., Chaisakul J. (2018). Non-neurotoxic activity of Malayan krait (*Bungarus candidus*) venom from Thailand. J. Venom. Anim. Toxins Incl. Trop. Dis..

[36] Sachdeva J., Dai W., Kloner R.A. (2014). Functional and histological assessment of an experimental model of Takotsubo’s cardiomyopathy. J. Am. Heart Assoc..

[37] Nanayakkara D.P., Ratnayake R.M.P., Shirani Ranasinghe J.G. (2009). Histopathological Changes in Brain, Kidney and Liver of Mice following Intramuscular Administration of Krait Venom. Cey. J. Sci. (Bio. Sci.).

