# Supplementary material for: Histopathological Changes in the Liver, Heart and Kidneys Following Malayan Pit Viper (Calloselasma rhodostoma) Envenoming and the Neutralising Effects of Hemato Polyvalent Snake Antivenom
Source: Toxins (Basel). 2022 Aug 30;14(9):601. doi: 10.3390/toxins14090601 (PMC9505761; doi:10.3390/toxins14090601)
Supplement: Supplementary file 1 [file toxins-14-00601-s001.zip › toxins-1864829-supplementary.pdf]

Supplementary Materials: Histopathological Changes in the Liver, Heart and Kidneys Following Malayan Pit Viper (*Calloselasma rhodostoma*) Envenoming and The Neutralising Effects of Hemato Polyvalent Snake Antivenom

Wipapan Khimmaktong, Nazmi Nuanyaem, Nissara Lorthong, Wayne C. Hodgson and Janeyuth Chaisakul

Table S1. Pathological characteristic and evaluation criteria for degree of morphological changes in liver [37].

Tissue change	Description	Score
Congestion	• No congestion	0
	• In few sinusoids and vessels	+
	• In about half of sinusoids and vessels	++
	• In almost all sinusoids and vessels	+++
Inflammatory infiltration	• No inflammatory infiltrate	0
	• 1-3 inflammatory foci/ section	+
	• 4-6 inflammatory foci/ section	++
	• >6 inflammatory foci/ section	+++
Necrosis	• No necrosis	0
	• Focal necrosis	+
	• Zonal necrosis	++
	• Confluent necrosis	+++

Table S2. Pathological characteristic and evaluation criteria for degree of morphological changes in kidneys [37].

Tissue change	Description	Score
Congestion	• No congestion	0
	• Focal glomeruli and interstitial vessels	+
	• Diffuse glomeruli and interstitial vessels	++
	• In almost all glomeruli and interstitial vessels or hemorrhage	+++
Inflammatory infiltration	• No inflammatory infiltrate	0
	• 1-3 inflammatory foci/ section	+
	• 4-6 inflammatory foci/ section	++
	• >6 inflammatory foci/ section	+++
Tubular injury	• No evidence of tubular injury	0
	• Less than 50% loss of brush border	+
	• More than 50% loss of brush border with cell sloughing off	++
	• Tubular necrosis is found in most area	+++

Table S3. Pathological characteristic and evaluation criteria for degree of morphological changes in heart [36].

Tissue change	Description	Score
Myocardial Damage	• No lesions	0
	• Slight derangement of muscle fibers, few inflammatory cells and vacuoles	0.5
	• Focal lesions of the subendocardial portion of the apex and mid-ventricle, inflammatory cells, interstitial edema, vacuolization of myocytes	1
	• Focal lesions of the subendocardium of the apical and mid ventricular region with right ventricular involvement	1.5
	• Focal lesions extending over a wider area of both ventricles	2
	• Focal lesions extending over a wider area of both ventricles, extensive inflammatory cell infiltration, interstitial edema, rupture of myofibers	2.5
	• Confluent lesions of the apex, mid-left ventricle and right ventricle, extensive inflammatory cell infiltration, profuse edema	3
	• Confluent lesions throughout the heart	4
